# A Randomized Controlled Trial of the Effect of 0.01% Atropine Eye Drops Combined with Auricular Acupoint Stimulation on Myopia Progression

**DOI:** 10.1155/2021/5585441

**Published:** 2021-08-10

**Authors:** Xie-He Kong, Yue Zhao, Zhi Chen, Li Zeng, Rong Han, Xiao-Qing Dong, Xiao-Cong Guo, Zheng Shi, Guang Yang, Yan-Ting Yang, Dan Zhang, Xing-Tao Zhou, Xiao-Peng Ma

**Affiliations:** ^1^Shanghai Research Institute of Acupuncture and Meridian, Shanghai 200030, China; ^2^Department of Ophthalmology and Visual Science, Eye and ENT Hospital of Fudan University, Shanghai 200031, China; ^3^NHC Key Laboratory of Myopia, Fudan University, Shanghai 200031, China; ^4^Laboratory of Myopia, Chinese Academy of Medical Sciences, Shanghai 200031, China; ^5^Shanghai University of Traditional Chinese Medicine, Shanghai 201210, China

## Abstract

**Background:**

Use of 0.01% atropine eye drops (0.01% A) is one of the most common treatments for myopia control for children in Asia. Auricular acupoint stimulation (AAS) was reported to enhance the effect of higher-concentration atropine (0.25%, 0.125%) on myopia control. This study was designed to compare the effect of 0.01% A combined with AAS and 0.01% A alone on myopia progression and choroidal thickness in children.

**Methods:**

A total of 104 children were stratified by age and randomly assigned at 1 : 1 to receive 0.01% A or 0.01% A + AAS treatment for 6 months. Repeated measurements of cycloplegic spherical equivalent (SE) autorefraction, axial length (AL), and choroidal thickness were performed at baseline, 1 month, 3 months, and 6 months.

**Results:**

The adjusted mean SE change over the 6 months was −0.38 ± 0.04 D in the 0.01% A group (*n* = 50) and −0.25 ± 0.04 D in the 0.01% A + AAS group (*n* = 50), demonstrating a significant between-group difference (*P* = 0.02). There was no statistically significant difference in the change of AL and choroidal thickness between the two groups (both *P* > 0.05).

**Conclusions:**

Adjunctive AAS compared with 0.01% A monotherapy slowed myopic progression in Chinese children by a statistically small amount, but had no effect on axial elongation and choroidal thickness during this 6-month observation. The trial is registered with ChiCTR1900021316.

## 1. Introduction

The global prevalence of myopia is increasing rapidly in recent decades, predominantly in East Asia. Large-scale investigations conducted in China indicated that the prevalence of myopia is now 70% to 85% in students at the age of 17 to 18 [1–3], being much higher than that of 20% to 40% seen in many Western countries [4–6]. High myopia predisposes the patients to a number of severe ocular complications, such as retinal detachment and macular degeneration, leading to loss of vision and blindness [7]. Therefore, myopia prevention and control has become an important issue to be solved urgently.

Acupoint stimulation is a traditional Chinese medicine therapy that can regulate whole body function to achieve a therapeutic effect. Chinese eye exercises have been performed by school children in China as an “acupoint pressing” intervention for the purpose of reducing ocular fatigue and preventing myopia for around 60 years, but the efficacy is still a matter of debate [8–10]. Acupuncture, as a promising acupoint stimulation measure, has long been used in ophthalmology diseases [11]. Shang et al. [12] showed that acupuncture reduced −0.07 D myopia progression in a 5-week treatment, but the results were limited by small sample size and lack of a blank control. The efficacy of acupuncture depends on continuous treatment 2 to 3 times per week, which is less acceptable for children with heavy academic burden and fear of needles. Auricular acupoint stimulation (AAS) may be a suitable choice for preventing myopia in children because of its simple and noninvasive operation. Two studies [13, 14] showed that auricular acupoint pressing could temporarily improve the unaided visual acuity and delay the progression of myopia compared with the blank control group, but the results were calculated based on noncycloplegic refraction, and the observation periods were short.

In recent years, administration of 0.01% atropine eye drops (0.01% A) has become one of the most common treatment modalities for myopia control for children in Asia [15]. Compared with blank control and placebo control, 0.01% A could slow myopia progression by roughly 0.25 D in one year [16, 17]. In contrast, the effect of 0.01% A on axial length (AL) elongation remains controversial [16–18]. Therefore, a higher-concentration atropine has been intended for children who showed poor response to 0.01% atropine. In LAMP2 study [19], the efficacy of 0.05% atropine on myopia control was twice that of 0.01% atropine. However, 0.05% atropine induced 2 D accommodative amplitude loss and 1.25 mm pupil dilation, not favorable in terms of long-term safety. Since AAS has been shown to enhance the effect of higher-concentration atropine (0.25% [20] and 0.125% [21]) on myopia control, the question remains as whether AAS might also enhance the effect of 0.01% A without inducing significant side effects.

Being an integral part of the mechanisms underlying myopia onset and development, the choroid has been extensively explored in the last decade [22, 23]. Scleral hypoxia caused by choroidal thinning has been hypothesized to result in myopia progression [24]. Since AAS was reported to alter the blood flow velocity of ophthalmic artery [25], we speculate that AAS can promote the blood flow of choroid, which may in turn thicken the choroid and produce curative effect on myopia progression.

The purpose of the present study was to evaluate the effect of 0.01% A combined with AAS and 0.01% A alone on myopia progression and choroidal thickness in children.

## 2. Materials and Methods

### 2.1. Study Design

This study was a prospective, randomized, assessor- and statistician-masked, controlled trial comparing the effect of 0.01% A + AAS and 0.01% A alone on myopia progression and choroidal thickness in Chinese children. The protocol and informed consent were approved by the Institutional Ethical Committee Review Board of Fudan University Eye & ENT Hospital and also was registered with the Chinese Clinical Trial Registry (identifier: ChiCTR1900021316). All subjects were treated in accordance with the tenets of the Declaration of Helsinki.

### 2.2. Eligibility Criteria

The study recruitment was announced via an official account on social media. The guardians of the subjects were instructed to read the informed consent and those who responded positively were scheduled for the screening visit. Inclusion criteria were aged 7 to 12 years with myopic refraction between −6.00 D and +0.50 D, astigmatism of less than 1.50 D, anisometropia of less than 1.50 D, and intraocular pressure between 10 and 21 mmHg. Excluded were those who had other ocular diseases (e.g., cataract, uveitis, and amblyopia), auricular diseases or systemic diseases, allergy to atropine, previous use of atropine, or any other myopia control treatment within 1 month. Informed consent was obtained from both subjects and their guardians.

### 2.3. Randomization and Masking

In a permuted block design stratified by age (7 to 9 and 10 to 12), each subject was randomly assigned with equal probability to receive 0.01% A treatment or 0.01% A + AAS treatment. The randomization sequence was generated by a third party using SPSS 24.0. The assessors and the statisticians were masked to the treatment allocation, while open to the acupuncturists. In the 0.01% A group, subjects and their guardians were told to remove the magnetic plasters before each assessment and not to discuss any issues related to auricular acupoint with the assessors.

### 2.4. Interventions

**Figure 1:**
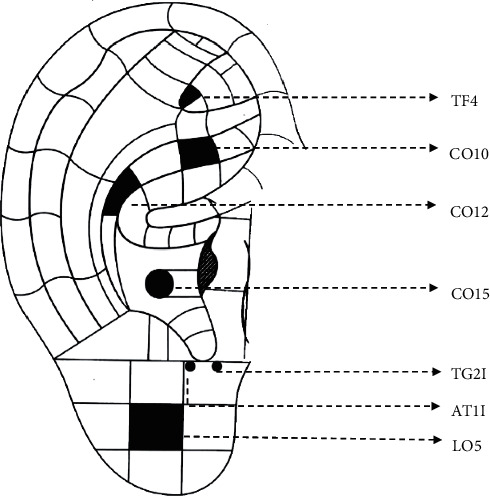
Selected auricular points for preventing myopia. This picture was modified according to the auricular map issued by the World Federation of Acupuncture-Moxibustion Societies [26].

Subjects in 0.01% A group were treated with a single drop of 0.01% atropine eye drops (0.4 ml:0.04 mg, Shenyang Xingqi Pharmaceutical Co., Ltd., Shenyang, China) every night for 6 months. Subjects in 0.01% A + AAS group received 0.01% atropine treatment plus auricular acupoint stimulation for 6 months. Seven auricular acupoints on the ear, including Eye (LO5), Anterior Intertragic Notch (TG2l), Posterior Intertragicus (AT1l), Heart (CO15), Liver (CO12), Kidney (CO10), and Shenmen (TF4) were selected (see Figure 1) according to traditional Chinese medicine theory based on review of the literature [14, 20, 21]. A magnetic bead plastered with 7 mm tape (Hwato, Suzhou Medical Appliance Factory Co., Ltd., Suzhou, China) was used for acupoint stimulation. Pressing stimulation was administered 3 times per day (6 : 30–7:30, 15 : 30–16 : 30, and 20 : 00–21 : 00) and 30 times for each acupoint. In order to avoid the decrease of acupoint sensitivity and skin allergy, only unilateral auricles were pressed every week, and bilateral auricles were carried out alternately. The acupoint plaster was changed weekly by licensed acupuncturists with more than 2 years' experience. At the same time, the teaching videos and maps describing AAS therapy were distributed to the guardians for learning, so that the magnetic beads fell from the acupoints by chance (e.g., bathing) could be pasted in time. Quality control was performed by the acupuncturists through checking the instant photos sent by the guardians.

### 2.5. Outcome Measures

After the screening visit (baseline), subjects were reassessed at 1, 3, and 6 months. At each visit, spherical equivalent (SE), axial length (AL), and choroidal thickness were measured.

Cycloplegic autorefraction was measured using an open-field autorefractor (WAM-5500, Grand Seiko Ltd., Japan) [27] 30 minutes after one drop of 0.5% Alcaine and two drops of 1% cyclopentolate HCL in 5-minute interval. This equipment allowed a sensitivity of 0.01 D, which could detect subtle diopter differences. The subjects were instructed to fixate on a Maltese cross at 5 m during measurements. If the subject's uncorrected visual acuity was <6/12, a green spotlight was utilized to minimize eye movements. SE was calculated for autorefraction readings. Five SE outcomes were obtained and averaged.

AL was measured by a swept source optical coherence tomography based IOL-Master 700 (Carl Zeiss Meditec, Inc., Germany) [28]. Three readings of repeated measurements and difference no greater than 0.02 mm were taken and averaged.

Images of the choroid in the macular region were obtained using a spectral domain OCT (RS-3000, NIDEK, Co., Ltd., Japan). This OCT device uses a superluminescent diode of central wavelength 880 nm for OCT imaging, with an axial resolution of 7 *μ*m and transverse resolution of 20 *μ*m. A three-dimensional scanning procedure was performed with a 6 × 6 mm raster scan centered on the fovea, which was composed of 128 B-scans. Each image is the average of 10 scans and images with motion artifacts, blinking, or segmentation failure were not included in the data analysis.

### 2.6. Additional Measures

This study design also included an evaluation of changes in anterior chamber depth (ACD) and intraocular pressure (IOP) at each timepoint.

The ACD was measured using IOL-Master 700. IOP was measured by a noncontact tonometer (NT-510, NIDEK, Co., Ltd., Japan). Three measurements were taken and averaged.

### 2.7. Data Analysis

The analysis for OCT imaging in terms of choroidal volume was processed in three steps: attenuation compensation, choroid segmentation, and en face mapping. The attenuation compensation algorithm was proposed [29] to enhance the visibility of the sclera-choroid interface and to minimize the projection shadows of retinal vessels in previous publications [30, 31]. We then employed the U-shape convolutional network (U-Net) [32, 33] to automatically segment the choroid in OCT. The trained U-Net for the choroid segmentation here achieved an AUSDE of 2.65 pixels. We deployed it to segment the data used in this paper and manually checked the results afterwards. The segmentation results were used to calculate the subfoveal choroidal thickness (SChT) and average choroidal thickness (AChT) by manually localizing the fovea and averaging over the entire 6 × 6 mm^2^ field of view, respectively. More methodological details are shown in Additional File 1.

### 2.8. Statistical Analysis

Based on a previous study [20], the enhanced effect of AAS was assumed to be at least 0.17 D, with a common standard deviation of 0.28 D in each treatment group. The sample size was estimated to be 88 (44 per group) to achieve 80% power at a 0.05 significance level (two-sided). Considering a 10% nonadherence to treatment and a 5% loss to follow-up, a sample size of 104 subjects (52 per group) would be needed.

Full analysis set was performed based on the intention-to-treat principle. Since no statistically significant differences were found in baseline data between the two eyes, only data from right eyes were used for statistical analyses. Baseline characteristics between the two treatment groups were evaluated by unpaired *t*-tests for continuous data meeting normality assumptions and the chi-square test or the Fisher exact test for categorical data.

Generalized estimating equations (GEE), with one within-subject factor (time), one between-subject factor (treatment: 0.01% A or 0.01% A + AAS) and their interactions, were used to compare changes in all outcomes. Age, sex, baseline SE, number of myopic parents, and outdoor time were included in the GEE model to determine the changes of SE and AL in the two study groups. Bonferroni's adjustments were used for pairwise comparisons. Pearson's correlation tests were used to evaluate the association between changes in SE and AL. We undertook ancillary analyses to confirm the treatment effects across potentially prognostic subgroups. These subgroups were sex, age, and baseline SE. The models also included the subgroup as a factor and the interaction between treatment and subgroup to test the significance of any difference in treatment effects across the subgroups. The significance level was set at *P* < 0.05.

## 3. Results

**Figure 2:**
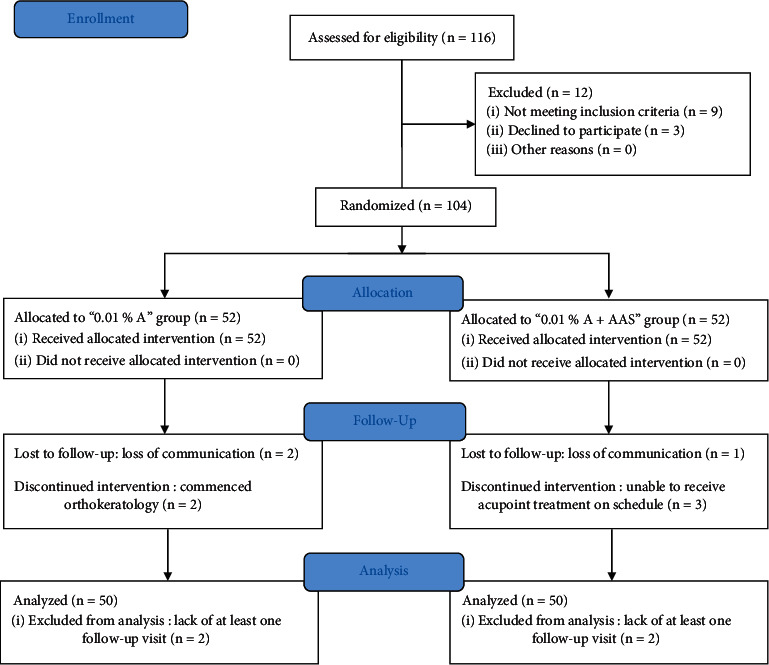
Schematic diagram showing the study flow.

Figure 2 represents the flowchart of the study. A total of 116 subjects were assessed for eligibility, in which 104 subjects were recruited into the study, with 52 subjects allocated to the 0.01% A group and 52 subjects to the 0.01% A + AAS group. Eight subjects did not complete all the follow-up visits, with 4 in the 0.01% A group and 4 in the 0.01% A + AAS group. Among these eight subjects, four did not receive any assessment after baseline, so their data were excluded from the analysis. Two subjects were unable to receive AAS treatment on schedule and the measurements at their final follow-up visits were carried forward. Two subjects commenced orthokeratology after three months' follow-up visit, and their measurements at three months were carried forward. There was no statistically significant difference among groups in baseline characteristics of the two groups (all *P* > 0.05) (Table 1). The atropine use was 6.44 times per week (92%) in the 0.01% A group and 6.34 times per week (90.6%) in the 0.01% A + AAS group, respectively. The implementation of AAS was 2.56 times per day (85.3%). Both groups showed good compliance during treatment (>85% expected use).

### 3.1. Change in SE

The tests of model effect indicated that treatment, time, and age (all *P* < 0.05) had significant association with the magnitude of SE change.

**Figure 3:**
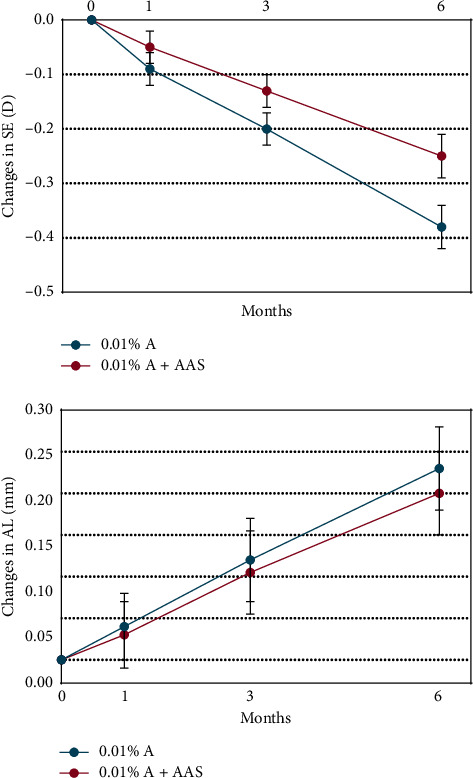
Adjusted mean change of myopia progression from baseline to 6 months. SE: spherical equivalent; AL: axial length.

The adjusted mean SE change over 6 months (Table 2 and Figure 3) was −0.38 ± 0.04 D in the 0.01% A group (*n* = 50) and −0.25 ± 0.04 D in the 0.01% A + AAS group (*n* = 50), being significantly different between groups (mean differences 0.13 ± 0.06 D, *P* = 0.02). Controlling for covariates did not significantly change the result (*P* = 0.02).

The 6-month change in SE stratified by baseline characteristics is shown in Table 3. Compared with the 10 to 12-year age group (−0.23 D in 0.01% A and −0.17 D in 0.01% A + AAS), the 7- to 9-year age group showed more myopic progression, with or without adjunctive AAS (−0.47 D in 0.01% A and −0.29 D in 0.01% A + AAS). As indicated by the interaction terms from the model analyses, the enhancement effect of AAS was not related to age (*P* = 0.30), sex (*P* = 0.70), or baseline SE (*P* = 0.07). However, compared with subjects at the age of 10 to 12, those who were at the age of 7 to 9 had a significantly less SE change in the 0.01% A + AAS group than in the 0.01% A group (mean differences: 0.18 ± 0.06 D, *P* = 0.01). Compared with subjects with baseline SE ≥ −2.25 D, those who had baseline SE < −2.25 D had a significantly less SE change in the 0.01% A + AAS group than in the 0.01% A group (mean differences: 0.25 ± 0.07 D, *P* < 0.001).

### 3.2. Change in AL

The tests of model effect indicated that time and age (both *P* < 0.05) had significant association with the magnitude of AL increase.

The adjusted increase of AL over 6 months (Table 2 and Figure 3) was 0.23 ± 0.05 mm in the 0.01% A group (*n* = 50) and 0.20 ± 0.05 mm in the 0.01% A + AAS group (*n* = 50), with no significant differences between groups (*P* = 0.15). Controlling for covariates did not significantly change the result (*P* = 0.19).

AL elongation over 6 months stratified by baseline characteristics is shown in Table 3. Results of AL within different age groups were similar to those reported for SE. The 7- to 9-year age group showed more axial elongation with or without adjunctive AAS (0.27 mm in 0.01% A, 0.22 mm in 0.01% A + AAS) when compared with the older age group (0.19 mm in 0.01% A, 0.17 mm in 0.01% A + AAS). Interaction analyses revealed that the effect of AAS treatment was not correlated to age (*P* = 0.61), sex (*P* = 0.13), or baseline SE (*P* = 0.09). However, significant differences between groups were observed in subjects with baseline SE < −2.25 D (mean differences −0.08 ± 0.03 mm, *P* = 0.02).

Overall, the Pearson correlation coefficient between the change in AL and the change in SE was 0.78 for the 0.01% A group and 0.81 for the 0.01% A + AAS group (both *P* < 0.001).

### 3.3. Change in Choroidal Thickness

The tests of model effect indicated that only time had a significant association with SChT (*P* = 0.033) and AChT (*P* = 0.039) (Table 2). A relatively small amount of SChT thinning at 6 months was found in both the 0.01% A group (−9.18 ± 2.76 *μ*m, *P* < 0.001) and the 0.01% A + AAS group (−7.92 ± 2.92 *μ*m, *P* < 0.001) while AChT thinning at 6 months was only found in the 0.01% A group (−4.75 ± 2.04 *μ*m, *P* = 0.033) after Bonferroni's correction. However, no differences in SChT (*P* = 0.55) or AChT (*P* = 0.45) were observed between groups.

### 3.4. Change in ACD and IOP

Time was the only factor that had a significant correlation with ACD (*P* < 0.001) (Table 2). ACD was significantly increased in both groups, but there was no statistically significant difference in the mean change of ACD between the two groups at any timepoints (all *P* > 0.05). No significant between- or within-group differences were detected in IOP (all *P* > 0.05).

## 4. Discussion

In this randomized clinical trial, we found a statistically significant 6-month enhancement effect of adjunctive AAS (*P* = 0.02), with an adjusted mean difference of 0.13 D in SE change between the 0.01% A + AAS and the 0.01% A group. This difference was clinically negligible, yet statistically significant. In concordance with our finding, Liang et al. [20] found that the adjunctive AAS treatment with 0.25% atropine eye drops slowed myopia progression by 0.17 D, and Chen et al. [21] found that the adjunctive AAS treatment with 0.125% atropine eye drops slowed myopia progression by 0.25 D over 12-month follow-up. Surprisingly, there was no significant difference in AL change between the two groups in the current study. The positive results in SE cannot be attributed to ACD because no significant difference in the change of ACD was seen between the two groups. Since the change in SE was highly correlated with changes in AL and the correlation coefficients were almost similar between the two groups (0.78 in the 0.01% A group and 0.81 in the 0.01% A + AAS group), it remains unclear why there is a mismatch between the two parameters. Our sample size may be too small and the observational span too short to detect the AL difference between the two groups. We speculate that the enhancement effect may continue to accumulate over the next 6 months.

Given that age is a vital influencing factor for myopia progression [34], we stratified the randomization by the age of subjects. Our results also confirmed that age had a significant correlation with the magnitude of SE and AL change. Participants aged between 7 and 9 years experienced more myopia progression than those aged between 10 and 12 years, with or without adjunctive AAS. Ancillary analyses showed that children with higher myopia or lower age might benefit more from AAS as an adjunctive treatment. This phenomenon may be explained by the theory of “acupoint sensitization” [35] that the status of acupoints can switch from a “silent” to “active” state during pathological processes and stimulating sensitized acupoints can exert a better effect. According to Donovan's study [36], myopia progression declines from −1.12 D/y at 7 years old to −0.50 D/y at 12 years old among Asian children. Additionally, more myopic baseline refractive error was related to higher risk of myopia [37] and high myopia [38] onset. Therefore, the myopia-related acupoints of children in these two subgroups were likely to be more active, which might be the reason why they benefited more from the combined AAS treatment.

Previous studies have suggested that choroid may play a bridging role in the signal cascades of myopia development. A bidirectional change of choroidal thickness in response to different retinal defocus signals was found in both human eyes and animal models [39–41]. Thinning of the choroid may lead to scleral hypoxia, which promote scleral extracellular matrix remodeling and myopia ensues consequently [24]. In the current study, we did not find a significant effect of AAS treatment on SChT or AChT at 1 month, 3 months, or 6 months. This suggests that the choroidal thickness change is unlikely to act as a primary mechanism to promote the enhancement effect of AAS in slowing myopia progression. Further studies are needed to explore the underlying mechanism.

High-concentration atropine (1%, 0.5%) eye drops have been shown to increase choroidal thickness and eliminate the effect of hyperopic defocus on choroidal thinning in human eyes [42, 43]. In contrast, the impact of low-concentration atropine (0.01%) on choroidal thickness remains controversial. Studies with positive results revealed a much less choroidal thickening (e.g., 6 *μ*m after 1 hour [44] and 5 *μ*m after 1 month use of 0.01% A [45]). Since some children who experience fast AL growth will exhibit a thinning of the choroid [23], it is not surprising that we found a slightly thinner choroid after 0.01% A use for 6 months. The inconsistency between the previous studies and the current one could also be explained by the different OCT methodologies applied. We adopted an automatic segmentation method through machine deep learning as opposed to manual differentiation of the choroidal boundary as used by most of the other studies, which inevitably incurs human artifacts. The long-term impact of 0.01% A or combined with AAS on choroidal thickness warrants further investigation.

Cheng and Hsieh's study [21] denoted that children treated with AAS in combination with 0.125% topical atropine had more ACD increase and more IOP reductions than those treated with atropine alone. Different from those findings, our study found no significant impact of AAS on ACD and IOP. The high rate of dropout (63%) in Cheng and Hsieh's study might expose the published data to a systematic bias. The IOP in both groups remained stable during our study period, indicating that 0.01% A has no risk associated with elevated IOP as previously reported [17].

Our study has some limitations. First, we did not use sham acupoint plaster with no bead as adjunctive intervention in the 0.01% A group for placebo control. Since AAS is widely used in China and soreness caused by pressing is well recognized by many people, it is difficult to implement a sham intervention by using a bead-free acupoint plaster as a placebo. Second, this study has no blank control group without any pharmaceutical intervention. Therefore, the question as to whether the two treatment modalities applied in this study has any effect on myopia progression remains unanswered. Third, this study was limited by its small sample size, and studies with longer observational span and larger sample size are needed to illustrate whether the enhancement effect of AAS is sustainable.

## 5. Conclusions

To conclude, adjunctive AAS compared with 0.01% A monotherapy slows myopic progression in Chinese children but has no effect on axial elongation and choroidal thickness within 6 months of treatment. Children with higher myopia and lower age might benefit more from AAS as an adjunctive treatment.

## Figures and Tables

**Table 1:** Baseline characteristics of study subjects.

	0.01% A (*n* = 50)	0.01% A + AAS (*n* = 50)	*P*
Age (years)	9.12 ± 1.39	8.96 ± 1.38	0.57
Gender, *n* (%)			
Male	22 (44%)	28 (56%)	0.23
Female	28 (56%)	22 (44%)	
Intraocular pressure (mmHg)	15.22 ± 2.51	15.39 ± 2.58	0.74
Spherical equivalent (D)	−2.25 ± 1.14	−2.14 ± 1.27	0.64
Axial length (mm)	24.48 ± 0.76	24.30 ± 0.86	0.25
Keratometry (D)	43.14 ± 1.53	43.46 ± 1.44	0.29
Anterior chamber depth (mm)	3.77 ± 0.21	3.78 ± 0.18	0.71
Lens thickness (mm)	3.34 ± 0.15	3.36 ± 0.14	0.48
Subfoveal choroidal thickness (*μ*m)	233.45 ± 22.95	233.83 ± 28.68	0.95
Average choroidal thickness (*μ*m)	219.94 ± 17.94	223.00 ± 19.59	0.46
Outdoor time (hours per week)^a^	8.14 ± 3.25	7.83 ± 3.46	0.48
Parental myopia, *n* (%)			
0	4 (8%)	2 (4%)	0.37
1	12 (24%)	18 (36%)	
2	34 (68%)	30 (60%)	

Values are expressed as means ± standard deviation unless stated otherwise. ^a^Cumulative time outdoors from 7:00 to 17:00 except cloudy and rainy days.

**Table 2:** Change in ocular parameters at different timepoints.

	0.01% A (*n* = 50)	0.01% A + AAS (*n* = 50)	*P* ^a^	*P* ^b^	*P* ^c^
*Spherical equivalent (D)* ^*d*^					
Change at 1 m	−0.09 ± 0.03	−0.04 ± 0.03	0.23	<0.001^*∗*^	0.36
Change at 3 m	−0.20 ± 0.03	−0.13 ± 0.03	0.12		
Change at 6 m	−0.38 ± 0.04	−0.25 ± 0.04	0.02^*∗*^		

*Axial length (mm)* ^*d*^					
Change at 1 m	0.04 ± 0.04	0.03 ± 0.04	0.37	<0.001^*∗*^	0.46
Change at 3 m	0.12 ± 0.05	0.10 ± 0.05	0.28		
Change at 6 m	0.23 ± 0.05	0.20 ± 0.05	0.15		

*Subfoveal choroidal thickness (μm)*					
Change at 1 m	−0.74 ± 2.55	2.09 ± 2.71	0.36	0.033^*∗*^	0.59
Change at 3 m	−3.15 ± 3.00	−1.57 ± 2.82	0.61		
Change at 6 m	−9.18 ± 2.76	−7.92 ± 2.92	0.55		

*Average choroidal thickness (μm)*					
Change at 1 m	−1.27 ± 1.60	0.04 ± 1.68	0.79		
Change at 3 m	−2.99 ± 1.87	−0.67 ± 1.69	0.21	0.039^*∗*^	0.61
Change at 6 m	−4.75 ± 2.04	−3.00 ± 1.70	0.45		

*Anterior chamber depth (mm)*					
Change at 1 m	0.01 ± 0.01	0.01 ± 0.00	0.76		
Change at 3 m	0.01 ± 0.01	0.02 ± 0.00	0.82	<0.001^*∗*^	0.48
Change at 6 m	0.03 ± 0.01	0.03 ± 0.01	0.28		

*Intraocular pressure (mmHg)*					
Change at 1 m	−0.27 ± 0.32	0.19 ± 0.29	0.33		
Change at 3 m	−0.11 ± 0.34	0.01 ± 0.33	0.80	0.17	0.18
Change at 6 m	0.93 ± 0.39	0.51 ± 0.37	0.48		

Values are expressed as means ± standard error. ^a^*P* value tests for group difference; ^b^*P* value tests for time; ^c^*P* value tests for the interaction between treatment and time; ^d^adjusted for age, sex, baseline spherical equivalent, number of myopic parents, and outdoor time. ^*∗*^Significant at 0.05.

**Table 3:** Adjusted 6-month change in myopia progression stratified by baseline characteristics.

Baseline characteristics	0.01% A	0.01% A + AAS	*P* ^b^	*P* ^c^
Changes in spherical equivalent (D)^a^				
*Age (years)*				
7∼9	−0.47 ± 0.05 (31)	−0.29 ± 0.05 (35)	0.01^*∗*^	0.30
10∼12	−0.23 ± 0.05 (19)	−0.17 ± 0.07 (15)	0.8	

*Gender*				
Male	−0.42 ± 0.07 (22)	−0.27 ± 0.05 (28)	0.08	0.70
Female	−0.33 ± 0.05 (28)	−0.23 ± 0.07 (22)	0.22	

*Spherical equivalent (D)*				
≥−2.25	−0.38 ± 0.05 (27)	−0.31 ± 0.05 (32)	0.64	0.07
<−2.25	−0.38 ± 0.06 (23)	−0.13 ± 0.07 (18)	<0.001^*∗*^	

Changes in axial length (mm)^a^				
*Age (years)*				
7∼9	0.27 ± 0.03 (31)	0.22 ± 0.02 (35)	0.14	0.61
10∼12	0.19 ± 0.02 (19)	0.17 ± 0.04 (15)	0.57	

*Gender*				
Male	0.26 ± 0.03 (22)	0.21 ± 0.03 (28)	0.07	0.13
Female	0.21 ± 0.02 (28)	0.19 ± 0.03 (22)	0.63	

*Spherical equivalent (D)*				
≥−2.25	0.23 ± 0.02 (27)	0.23 ± 0.02 (32)	0.57	0.09
<−2.25	0.24 ± 0.03 (23)	0.16 ± 0.03 (18)	0.03^*∗*^	

Values are expressed as means ± standard error. ^a^Adjusted for age, sex, baseline spherical equivalent, number of myopic parents, and outdoor time unless stratified by that factor. ^b^*P* value tests for group difference. ^c^*P* value tests for the interaction between treatment and baseline characteristics. ^*∗*^Significant at 0.05.

## Data Availability

The data used to support the findings of this study are included within the article.

## Abbreviations

AAS: Auricular acupoint stimulation
ACD: Anterior chamber depth
AChT: Average choroidal thickness
AL: Axial length
IOP: Intraocular pressure
LAMP: Low-concentration atropine for myopia progression
SChT: Subfoveal choroidal thickness
SE: Spherical equivalent
0.01% A: 0.01% atropine eye drops.

## Supplementary Materials

Supplementary Materials See Additional File 1 in the Supplementary Materials for understanding attenuation compensation and choroid segmentation.
