# Supplementary material for: A Randomized Controlled Trial of the Effect of 0.01% Atropine Eye Drops Combined with Auricular Acupoint Stimulation on Myopia Progression
Source: J Ophthalmol. 2021 Aug 10;2021:5585441. doi: 10.1155/2021/5585441 (PMC8373503; doi:10.1155/2021/5585441)
Supplement: Supplementary Materials — See Additional File 1 in the Supplementary Materials for understanding attenuation compensation and choroid segmentation. [file 5585441.f1.docx]

Supplementary file

*S1. Attenuation compensation*

Girard *et al.* developed an attenuation compensation algorithm to remove the OCT vessel shadows and enhance the contrast of optic nerve head [1]. This algorithm was then employed in the calculation of the attenuation coefficients of retinal tissue [2], enhancing the visibility of lamina cribrosa [3], and improving the contrast of the choroid vasculature and the visibility of the sclera-choroid interface [4,5]. It can be expressed as:

$I_{AC}\left( x,y \right)=\frac{I\left( x,y \right)}{2\sum_{k=x}^{M} I\left( k,y \right)},$ (1)

where *I_AC_* is the AC-enhanced image intensity and *I* is the original image intensity. $(x,y)$ are the pixel coordinates of the B-scan images ($x\in[1, M], y\in[1, N-1]$). *M* and *N* are the row and column numbers.


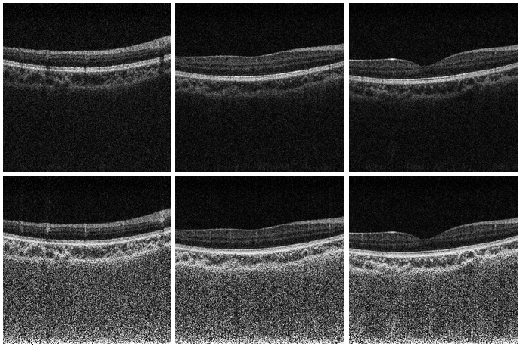


Figure 1 Examples of using attenuation compensation. Upper: Original B-scans. Lower: Enhanced B-scans.

*S2. Choroid segmentation*

We employ the U-shape convolutional network (U-Net) [6] to automatically segment the choroid in OCT. As shown in Fig. 2, the U-Net a fully convolutional network that includes convolution (Conv 3×3 and Up conv 2×2), max pooling (Max pooling 2×2), and nonlinear activations (ReLU and Sigmoid). It uses an Encoder path (left) to extract features and a symmetric Decoder path (right) to precise localization. To get better precise locations, at every step of the decoder, the U-Net uses skip connections by concatenating the output of the transposed convolution layers with the feature maps from the Encoder at the same level. The U-Net has achieved tremendous success in various medical image and semantic segmentation tasks. It enables high segmentation accuracy with limited sample sizes [6,7].


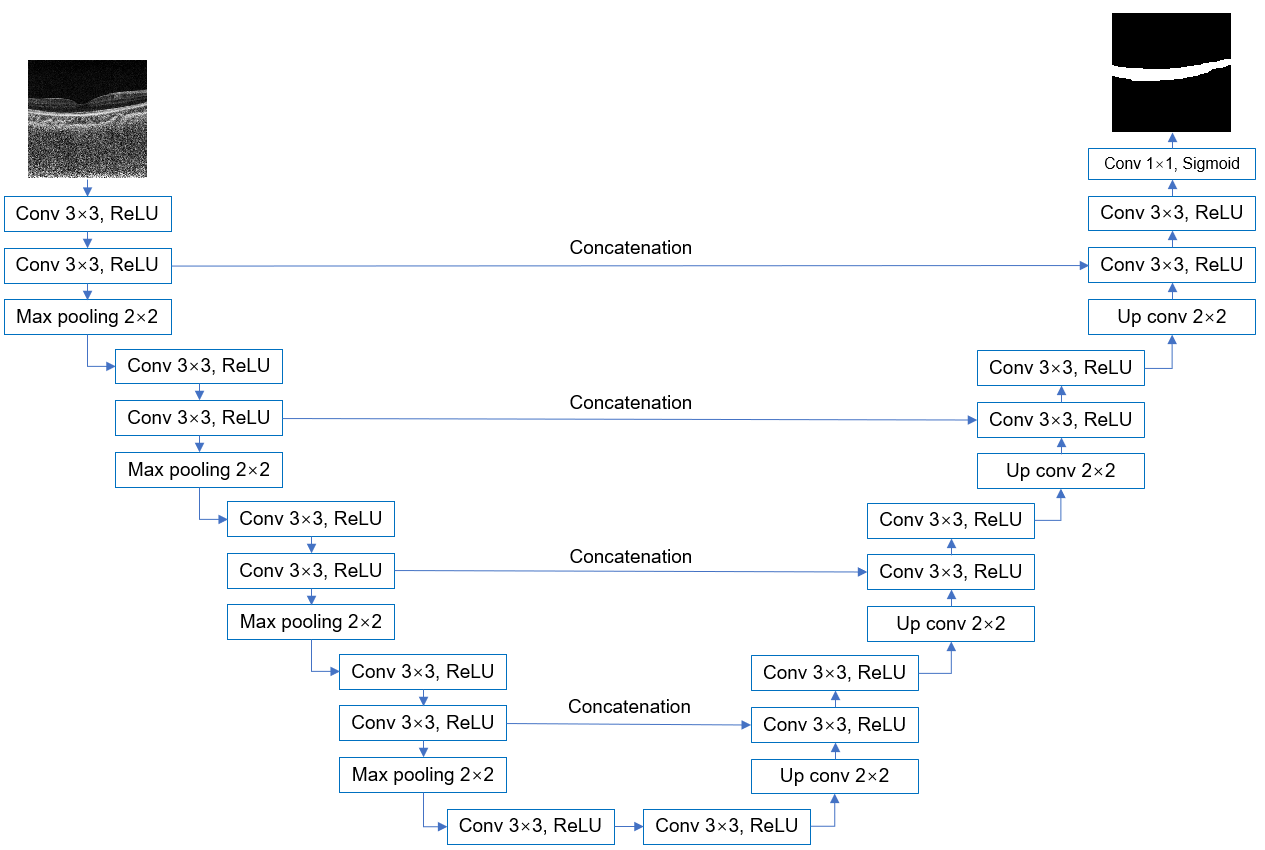


Figure 2 The U-shape convolutional neural network (U-Net) employed in the automatic segmentation of the choroid.

The U-Net for the choroid segmentation is implemented in Pytorch. We train the deep network with a total of 145 manually-annotated OCT B-scans. The training, validation, and testing sets are split with a ratio of 18:6:5. We employ the Adam optimizer with an initial learning rate of 10^-5^. We train a total of 200 epochs for the convergence of the model. We employ the average unsigned surface detection error (AUSDE) to quantitatively evaluate the segmentation performance, which calculates the pixel-wise mismatch between the segmented choroid boundary and the manual ground truth. We achieve an AUSDE of 2.65 pixels using the test set. We then deploy the trained model to segment the data used in this paper. After the automatic segmentation, we manually check the results frame by frame. Figure 3 is the examples of segmented choroid using U-Net. Upper: Input B-scans. Lower: the corresponding segmentation results. We can see the automatic segmentation is accurate for different positions of the 6×6 mm^2^ macular scans.


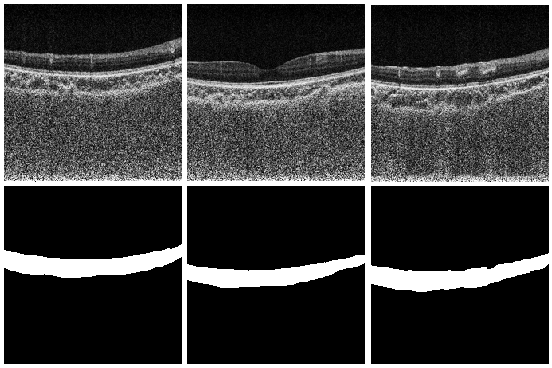


Figure 3 Examples of segmented choroid using U-Net. Upper: Input B-scans. Lower: the corresponding segmentation results.

**References:**

1. M. J. Girard, N. G. Strouthidis, C. R. Ethier, and J. M. Mari, “Shadow removal and contrast enhancement in optical coherence tomography images of the human optic nerve head,” Investigative ophthalmology & visual science, vol. 52, no. 10, pp. 7738–7748, 2011.
2. K. Vermeer, J. Mo, J. Weda, H. Lemij, and J. De Boer, “Depth resolved model-based reconstruction of attenuation coefficients in optical coherence tomography,” Biomedical optics express, vol. 5, no. 1, pp. 322–337, 2014.
3. J. M. Mari, N. G. Strouthidis, S. C. Park, and M. J. Girard, “Enhancement of lamina cribrosa visibility in optical coherence tomography images using adaptive compensation,” Investigative ophthalmology & visual science, vol. 54, no. 3, pp. 2238–2247, 2013.
4. H. Zhou, Z. Chu, Q. Zhang, Y. Dai, G. Gregori, P. J. Rosenfeld, and R. K. Wang, “Attenuation correction assisted automatic segmentation for assessing choroidal thickness and vasculature with swept-source oct,” Biomedical optics express, vol. 9, no. 12, pp. 6067–6080, 2018.
5. K. K. Vupparaboina, K. K. Dansingani, A. Goud, M. A. Rasheed, F. Jawed, S. Jana, A. Richhariya, K. B. Freund, and J. Chhablani, “Quantitative shadow compensated optical coherence tomography of choroidal vasculature,” Scientific reports, vol. 8, no. 1, p. 6461, 2018.
6. O. Ronneberger, P. Fischer, and T. Brox, “U-net: Convolutional networks for biomedical image segmentation,” in International Conference on Medical image computing and computer-assisted intervention. Springer, 2015, pp. 234–241.
7. N. Ibtehaz and M.S. Rahman, “MultiResUNet: Rethinking the U-Net architecture for multimodal biomedical image segmentation,” Neural Networks, 121, pp.74-87, 2020.
